# Diversity of Molecular–Network Conformations in the Over-Stoichiometric Arsenoselenides Covering a Full *Thioarsenides* Row As_4_Se_n_ (0 ≤ *n* ≤ 6)

**DOI:** 10.3390/molecules30091963

**Published:** 2025-04-29

**Authors:** Oleh Shpotyuk, Malgorzata Hyla, Zdenka Lukáčová Bujňáková, Yaroslav Shpotyuk, Vitaliy Boyko

**Affiliations:** 1 Institute of Physics, Jan Dlugosz University in Częstochowa, 13/15, al. Armii Krajowej, 42-200 Częstochowa, Poland; m.hyla@ujd.edu.pl; 2 O.G. Vlokh Institute of Physical Optics, Ivan Franko National University of Lviv, 23, Dragomanov Str., 79005 Lviv, Ukraine; 3 Scientific Research Company “Electron-Carat”, 202, Stryjska Str., 79031 Lviv, Ukraine; 4 Institute of Geotechnics of Slovak Academy of Sciences, 45, Watsonova Str., 04001 Košice, Slovakia; bujnakova@saske.sk; 5 Department of Sensor and Semiconductor Electronics, Ivan Franko National University of Lviv, 107, Tarnavskoho Str., 79017 Lviv, Ukraine; yashpotyuk@gmail.com; 6 Institute of Physics, University of Rzeszow, 1, Pigonia Str., 35-959 Rzeszow, Poland

**Keywords:** thioarsenide-type As_4_Se_n_ molecules, molecular network clustering, arsenoselenides, amorphization, crystallization, polymorphic transformations

## Abstract

Molecular network conformations in the over-stoichiometric arsenoselenides of canonical As_x_Se_100−x_ system (40 ≤ x ≤ 100) covering a full row of thioarsenide-type As_4_Se_n_ entities (0 ≤ n ≤ 6) are analyzed with ab initio quantum-chemical modeling employing cluster-simulation code CINCA. Native (melt-quenching-derived) and nanostructurization-driven (activated by nanomilling) *polymorphic* and *polyamorphic* transitions initiated by decomposition of the *thioarsenide*-type As_4_Se_n_ cage molecules and incorporation of their remnants into a newly polymerized arsenoselenide network are identified on the developed map of molecular network clustering in a binary As-Se system. Within this map, compositional counter lines corresponding to preferential molecular or network-forming tendencies in the examined arsenoselenides are determined, explaining that network-crystalline conformations prevail in the boundary compositions corresponding to n = 6 and n = 0, while molecular-crystalline ones dominate inside the rows corresponding to n = 4 and n = 3. A set of primary and secondary equilibrium lines is introduced in the developed clustering map to account for inter-phase equilibria between the most favorable (regular) and competitive (irregular) *thioarsenide* phases. Straightforward interpretation of decomposition reactions accompanying induced crystallization and amorphization (reamorphization) in the arsenoselenides is achieved, employing disproportionality analysis of *thioarsenide*-type molecular network conformations within the reconstructed clustering map. The preference of network clustering at the boundaries of the As_4_Se_n_ row (at n = 6 and n = 0) disturbs inter-phase equilibria inside this row, leading to unexpected anomalies, such as absence of stable tetra-arsenic triselenide As_4_Se_5_ molecular-crystalline species; polyamorphism in mechanoactivated As_4_Se_n_ alloys (2 ≤ n ≤ 6); breakdown in the glass-forming ability of melt-quenching-derived arsenoselenides in the vicinity of tetra-arsenic biselenide As_4_Se_2_ composition; plastically and normally crystalline polymorphism in tetra-arsenic triselenide As_4_Se_3_-based *thioarsenides*, and so on.

## 1. Introduction

Arsenic selenide alloys As_x_Se_100−x_ (also referred to as *arsenoselenides*) are an important class of substances that can be easily stabilized in a vitreous state starting from high-entropy melt by, respectively, rapid quenching within the broad compositional domain around stoichiometric arsenic triselenide As_2_Se_3_ (corresponding to x = 40), and stretching downwards to glassy Se (x = 0) and upwards to over-stoichiometric As-bearing species in a glassy state with up to ~70–75 at. % of arsenic (40 ≤ x < 70–75), or a glassy-crystalline state beyond this composition (x ≤ 100) [1,2,3,4,5]. Because of the full saturation of covalent chemical bonding in such binary chalcogenide systems, where elemental As and Se constituents are respectively two- and three-fold coordinated [1], their compositions are often defined by mean coordination number (MCN), that is, the average number of covalent chemical bonds per atom (MCN = 2 + 0.01x) [1,2].

Under-stoichiometric Se-bearing As_x_Se_100-x_ alloys (0 ≤ x ≤ 40, 2.00 < MCN < 2.40) exhibit glassy conformations possessing layered- or chain-type network structures [3,4] insensitive to post-technological modification [6,7]. In contrast, over-stoichiometric As-bearing As_x_Se_100−x_ compounds (40 ≤ x ≤ 100 or 2.40 ≤ MCN ≤ 3.00) attract significant attention in the contemporary glass manufacturers’ community in view of their post-technological modification possibilities, revealed by the diversity of structural conformations that can be derived from *thioarsenide*-type As_4_Se_n_ entities (0 ≤ n ≤ 6) [6,7,8,9,10,11]. Herein, we employ the *thioarsenide* nomenclature considering the As_4_X_n_ molecules as derivatives from As_4_ tetrahedra (n = 0) modified by insertion of the n-th chalcogen atom X into one of six intramolecular (As-As) bonds, which was initially introduced for analysis of molecular packing and electron density distribution in an analogous As-S system [12,13,14,15].

Within a full row of such introduced *thioarsenide*-type As_4_Se_n_ molecular entities from As (tetra-arsenic As_4_, n = 0) to tetra-arsenic hexaselenide As_4_Se_6_ (n = 6), we fit in over-stoichiometric As-bearing As_x_Se_100-x_ species deviated from ‘pure’ As (corresponding to x = 100, MCN = 3.00) to arsenic triselenide As_2_Se_3_ (corresponding to x = 40, MCN = 2.40) equivalent to As_4_Se_6_. With respect to the equilibrium phase diagram of a binary As-Se system [16,17], there are two intrinsic molecular-crystalline species within this compositional domain, these being monoclinic tetra-arsenic tetraselenide, As_4_Se_4_ (equivalent to arsenic monoselenide, AsSe [18,19,20]), and orthorhombic tetra-arsenic triselenide, As_4_Se_3_ [21]. However, the boundary species within the As_4_Se_n_ *thioarsenides* row (n = 6 and n = 0) are composed by network-crystalline structures typical for 2D-layered arrangements of corner-shared AsSe_3/2_ pyramids in monoclinic As_2_Se_3_ [18,22] or honeycomb arrangements of chair-configurated As_4_ rings in both rhombohedral (grey or metallic α-As) and orthorhombic (black or semiconducting β-As) allotropes [23,24,25,26]. The crystalline conformation composed of individual tetrahedral As_4_ molecules characteristic of yellow or insulator γ-As is very unfavourable, existing only in a gaseous state [25,26].

It is worth noting that similar molecular-crystalline allotropes are possible in tetra-arsenic triselenide *thioarsenides* due to phase equilibria in the vicinity of the As_4_Se_3_ composition [16,17]. Thus, with respect to Blachnik and Wickel [27], the monoclinic α-As_4_Se_3_ existing at ambient temperature transforms under heating above 412 K into high-temperature orthorhombic α’-As_4_Se_3_ modification, and under heating above 447 K, the latter transforms in plastically crystalline β-As_4_Se_3_ and an amorphous substance of unknown composition, while only orthorhombic α’-As_4_Se_3_ phase could be stabilized in metastable form by conventional melt quenching. That is why *polymorphic* inter-crystalline transitions are expected in molecular As_4_Se_3_ [9], in addition to *polyamorphic* transitions in As_x_Se_100−x_ alloys within a whole glass-forming region (40 ≤ x < 70–75, 2.40 ≤ MCN < 2.70–2.75) [8,10,11].

As was suggested recently by Shpotyuk and co-workers [8,9,10,11], similar decomposition reactions are also expected in other As_4_Se_n_ *thioarsenides,* resulting in a rich variety of molecular-to-network transitions. Specifically, *polyamophic* transitions are expected in over-stoichiometric arsenoselenides between some network species (network-forming clusters, NFC) derived from thioarsenide-type molecules (referred to as molecular-forming clusters, MFC), these transformations being activated by high-energy mechanical milling (*nanomilling)* [8,9,10,11]. This specificity determines the governing tendency of molecular-to-network transitions in *thioarsenide*-type arsenoselenides.

The objective of this research is comprehensive analysis to systemize a full row of *thioarsenide*-type As_4_Se_n_ molecular and network entities in over-stoichiometric arseno-selenides As_x_Se_100−x_ (40 ≤ x ≤ 100; 2.40 ≤ MCN ≤ 3.00). The generalized approach, which allows mapping of *thioarsenide*-type As_4_Se_n_ MFC and NFC, will be developed, employing ab initio quantum-chemical modeling with cluster-simulation code CINCA (cation-interlinked network cluster approach) [28,29]. This research is grounded in molecular network disproportionality analysis of As_4_Se_n_ *thioarsenides* (n = 0, 1, 2 [10], n = 3 [9], n = 4 [8], and *n* = 5, 6 [11]). Most plausible scenarios of nanostructurization-driven *polymorphic* and *polyamorphic* transitions are justified for over-stoichiometric arsenoselenides, covering a full row of As_4_Se_n_ molecules and their network derivatives (0 ≤ n ≤ 6).

## 2. Results and Discussion

### 2.1. Mapping of Molecular- and Network-Type Clusters in As_x_Se_100−x_ Arsenoselenides (20 < x ≤ 100)

Let us examine a full row of *thioarsenide*-type As_4_Se_n_ MFC (0 ≤ n ≤ 6) and their derivatives (NFC) within over-stoichiometric As-bearing arsenoselenides As_x_Se_100−x_ (40 ≤ x ≤ 100; 2.40 ≤ MCN ≤ 3.00). In fact, the As_4_Se_n_ MFC are derivatives from the tetrahedral As_4_ cage molecule stabilized by insertion of the n-th Se atom instead of one of six As-As bonds [12,13,14,15], while the As_4_Se_n_ NFC can be derived from the respective MFC by breaking in available Se atom positions followed by insertion of the remainder in a newly polymerized network. Therefore, in the atomic clusters nomenclature [28,29], the NFC formed by *N* breaks in Se atom positions are denoted by the number of these breaks following the *thioarsenide* formula x*N*-As_4_Se_n_, while the iso-compositional parent MFC stabilized without breaking is labeled as x0-As_4_Se_n_. In reality, we deal with iso-compositional MFC and NFC obtained by H atom termination in the broken Se atom positions of *thioarsenide*-type As_4_Se_n_ molecule (MFC) due to Se-H bonds (if a few configurations are possible, these clusters differ by an additional digit at the second place). For comparison, the forming energies of As_4_Se_n_ MFC and NFC *E_f_* = (*E*_f_^Σ^)^av^ are averaged for all (4 + n) atoms and simplified with respect to the forming energy of a trigonal AsSe_3/2_ pyramid (−72.309 kcal/mol) [29].

**Figure 1:**
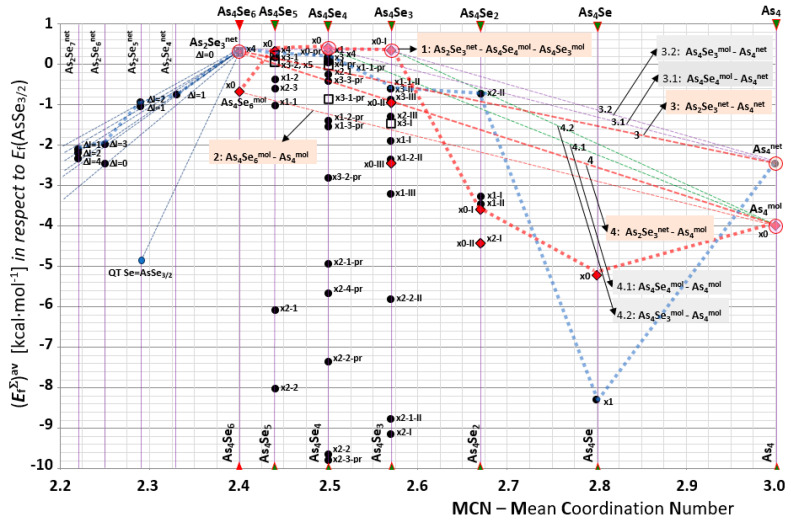
Compositional map of molecular network clustering in As_x_Se_100−x_ alloys (20 < x ≤ 100, 2.2 < MCN ≤ 3.0) covering full row of *thioarsenide*-type As_4_Se_n_ entities (0 ≤ n ≤ 6). The MFC are denoted by full red-colored rhombs, with ‘x0′ notation showing the absence of breaking in Se atom positions. Tho MFC that have crystalline counterparts are marked by open red circles. In the cases of a few MFC, they are additionally distinguished by -I, -II, or -III digits, while pararealgar-related As_4_Se_4_ MFC are labeled as ‘pr’. The NFC derived from As_4_Se_n_ MFC by N-fold breaking in Se atom positions are denoted by full black circles, with the right notation showing the number of breaks (xN). The NFC undergoing separation on a few parts are denoted by open black squares with the same indication of breaking. The dotted red-colored counter line connecting the settle-points of the most favorable *thioarsenide*-type MFC corresponds to the preference of the molecular-forming tendency in a system, while the network-forming tendency prevails along dotted blue-colored counter line connecting the settle-points of the most favorable NFC. The equilibrium lines numbered as 1, 2, 3, 3.1, 3.2, 4, 4.1, and 4.2 correspond to primary and secondary inter-crystalline phase equilibria in the binary As-Se system.

**Figure 2:**
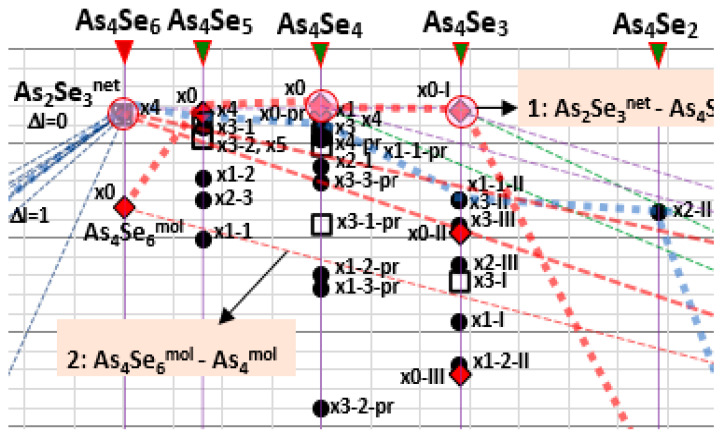
Fragment of compositional map of molecular network clustering in over-stoichiometric arsenoselenides reproduced within a range of glass-forming As_4_Se_n_ *thioarsenides* (2 ≤ n ≤ 6).

Compositional landscape mapping of all possible MFC and NFC in As_x_Se_100-x_ in a broad compositional domain around the As_2_Se_3_ stoichiometry (20 < x ≤ 100, 2.2 < MCN ≤ 3.0) covering a full row of *thioarsenide*-type As_4_Se_n_ entities (0 ≤ n ≤ 6) is reproduced in Figure 1, while a partial fragment of this map showing fine details of molecular network clustering within a range of glass-forming As_4_Se_n_ *thioarsenides* (2 ≤ n ≤ 6) is reproduced in Figure 2. The calculated *E*_f_ energies for iso-compositional MFC and NFC are gathered in Table 1.

In a group of under-stoichiometric Se-bearing As_x_Se_100−x_ alloys deviated from chemical As_2_Se_3_ stoichiometry (x = 40, MCN = 2.40) towards more Se-rich specimens (20 < x ≤ 40), we deal with two-cation As_2_Se_m_ NFC representing themselves as AsSe_3/2_ pyramidal units cross-linked by Se chains. Homogeneous linking with an equal number of Se half-atoms in all chains (Δl) corresponds to the canonical ‘chain-crossing’ model [2,3,4,5] (in the case of equivalent chains, Δl = 0 [29]). The diversity of As_2_Se_m_ NFC is defined by Δl parameters for each composition. Thus, going from stoichiometric As_2_Se_3_^net^ to Se-rich NFC through points corresponding to the most favorable entities (see Figure 1), we obtain a set of cut-section lines governing local (between neighboring NFC) and global (between terminated counterparts) decomposition reactions in under-stoichiometric arsenoselenides As_x_Se_100−x_ (x ≤ 40). It is worth noting that on the molecular network clustering map reproduced in Figure 1, we also denote the forming energy of a quasi-tetrahedral Se = AsSe_3/2_ unit based on double As = Se covalent bond [29], which seems to be far from the set of the lines ascribed to the As_2_Se_m_ NFC based on single As-Se bonds. This finding contradicts speculations that this optimally constrained quasi-tetrahedral unit can be considered as the principal glass-forming building block in the As_2_Se_5_ compound.

**Figure 3:**
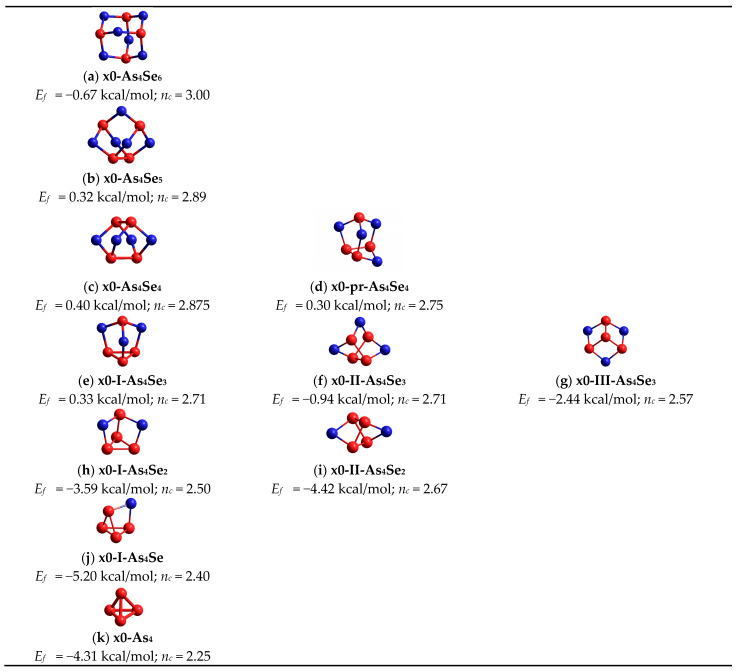
Ball-and-stick presentation of optimized configurations of *thioarsenide*-type As_4_Se_n_ MFC: (**a**) tetra-arsenic heksaselenide x0-As_4_Se_6_, (**b**) tetra-arsenic pentaselenide x0-As_4_Se_5_, (**c**) tetra-arsenic tetraselenide of realgar modification x0-As_4_Se_4_, (**d**) tetra-arsenic tetraselenide of pararealgar modification x0-pr-As_4_Se_4_, (**e**) tetra-arsenic triselenide in dimorphite-type triangular-pyramidal (As3)-As configuration x0-I-As_4_Se_3_, (**f**) tetra-arsenic triselenide in chain-like (As4) configuration x0-II-As_4_Se_3_, (**g**) tetra-arsenic triselenide in star-like (As-As3) configuration x0-III-As_4_Se_3_, (**h**) tetra-arsenic biselenide in combined triangular-star (As3)As configuration x0-I-As_4_Se_2_, (**i**) tetra-arsenic biselenide in chain-like (As4) configuration x0-II-As_4_Se_2_, (**j**) tetra-arsenic monoselenide x0-As_4_Se, (**k**) tetra-arsenic in tetragon-like configuration x0-As_4_. The Se and As atoms are blue- and red-colored, the bonds between atoms are denoted by respectively colored sticks, and the cluster-forming energies *E*_f_ are given in respect to the energy of AsSe_3/2_ unit (E_f_(AsSe_3/2_) = −72.309 kcal/mol [29]).

**Figure 4:**
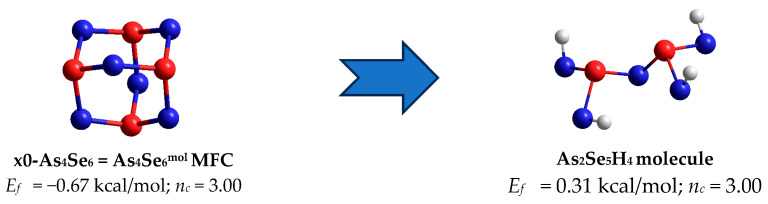
Ball-and-stick presentation of H-atom-saturated As_2_Se_5_H_4_ molecular prototype of the x4-As_4_Se_6_ = As_2_Se_3_^net^ NFC (on the **right**) derived from parent *thioarsenide*-type x0-As_4_Se_6_ = As_4_Se_6_^mol^ MFC (on the **left**) by breaking in four equivalent Se atom positions. The terminated H atoms are grey-colored; Se and As atoms are blue- and red-colored, respectively; and the bonds between atoms are denoted by respectively colored sticks.

In a group of tetra-arsenic hexaselenide *thioarsenide*-type As_4_Se_n_ entities (n = 6) corresponding to chemical stoichiometry in the As_x_Se_100−x_ system (that is, As_2_Se_3_ compositionally equivalent to As_4_Se_6_), the parent x0-As_4_Se_6_ MFC composed of four corner-sharing AsSe_3/2_ units in an optimally constrained topology (with the number of constraints per atom n_c_ = 3.00 strictly corresponding to space dimensionality, 3D) has very unfavorable cluster-forming energy of *E_f_* = −0.67 kcal/mol (see Table 1, Figure 1, Figure 2 and Figure 3a). The best energy, *E_f_* = 0.31 kcal/mol (with respect to the energy of a single AsSe_3/2_ pyramid [29]) is achieved for the NFC derived from this parent x0-As_4_Se_6_ MFC by breaking in four of six Se atom positions, labelled as x4-As_4_Se_6_ NFC (see Figure 4). This NFC can be imagined as two corner-sharing AsSe_3/2_ pyramidal units forming the topologically optimal layer-type network with n_c_ = 3.00 [11]. The calculated geometrical parameters of this x4-As_4_Se_6_ NFC, also labelled as As_2_Se_3_^net^ and denoted by an open circle in the compositional molecular network map (see Figure 1 and Figure 2), are found to be in very good agreement with those characteristic of the known crystalline counterpart, which is monoclinic As_2_Se_3_ [21,22].

Among tetra-arsenic pentaselenide As_4_Se_n_ entities (n = 5, MCN = 2.44), the parent x0-As_4_Se_5_ cage MFC composed of four small rings (2 pentagons, 2 hexagons) built of ten heteronuclear (As-Se) bonds and one homonuclear (As-As) bond in under-constrained topology (n_c_ = 2.89) possesses gradually improved *E*_f_ = 0.32 kcal/mol (see Figure 3b) [11]. The iso-compositional NFC stabilized as derivatives from this x0-As_4_Se_5_ MFC by breaking in Se atom positions (x4-As_4_Se_5_; x3-1-As_4_Se_5_; x3-2-As_4_Se_5_; x5-As_4_Se_5_) become competitive by their *E_f_* energies to parent x0-As_4_Se_5_ MFC (see Table 1, Figure 1 and Figure 2), preventing *thioarsenide*-type As_4_Se_5_ alloy from spontaneous crystallization [11].

In a group of tetra-arsenic tetraselenide As_4_Se_n_ entities (n = 4) with MCN = 2.50, the parent x0-As_4_Se_4_ MFC of *D*_2d_ symmetry (proper to As_4_S_4_ realgar [12,15]) is composed of eight small rings, resulting in n_c_ = 2.875 and the best *E_f_* energy among *thioarsenide*-type molecules, *E_f_* = 0.40 kcal/mol (see Table 1, Figure 1, Figure 2 and Figure 3c) [8]. This MFC is the main block of the molecular-crystalline counterpart (monoclinic As_4_Se_4_) [18,19,20]. The most plausible NFC of this type is the optimally constrained cluster (n_c_ = 3.00) stabilized by breaking in one of four equivalent Se atom positions (x1-As_4_Se_4_), which has three small rings, and over-constrained clusters with n_c_ = 3.25, such as x3-As_4_Se_4_ and x4-As_4_Se_4_ (see Table 1, Figure 1 and Figure 2). The former, with *E_f_* = 0.25 kcal/mol, seems competitive with x0-As_4_Se_4_ MFC, contributing to the optimal glass-forming ability of As_4_Se_4_ alloy [8,30]). The *nanomilling*-driven decrease in molecularity due to molecular-to-network transition essentially modifies the arrangement of diffuse peak-halos in the XRPD pattern of this alloy [7,8].

We do not exclude among As_4_Se_4_-based *thioarsenides* the possibility for pararealgar-type x0-pr-As_4_Se_4_ MFC and respective NFC [30]. By analogy with the pararealgar As_4_S_4_ molecule of C_s_ symmetry [12,15], the x0-pr-As_4_Se_4_ MFC (see Figure 3d) is composed of two homonuclear (As-As) bonds in neighboring geometry forming four small rings (1 hexagon, 2 pentagons, 1 tetragon), resulting in more under-constrained topology (*n_c_* = 2.75) as compared with realgar-type x0-As_4_Se_4_ MFC (Figure 3c). Despite the *E_f_* energy of x0-pr-As_4_Se_4_ MFC being competitive (0.30 kcal/mol), it has been still worse than this energy in x0-As_4_Se_4_ MFC, meaning an absence of pararealgar-type polymorph in the As-Se system. Nevertheless, this analysis does not disfavor x0-pr-As_4_Se_4_ MFC and its network derivatives, which could be stabilized in disordered materials such as glasses in non-equilibrium conditions of melt quenching [8,30]. With respect to modeling [30], the most favorable (apart from x0-pr-As_4_Se_4_ MFC) are optimally and over-constrained NFC, marked on the map of Figure 1 as x1-1-pr-As_4_Se_4_ (n_c_ = 3.0), x4-pr-As_4_Se_4_ (n_c_ = 3.25), and x3-3-pr-As_4_Se_4_ (n_c_ = 3.25).

There are three molecular-type conformations differing by their arrangement of four As atoms forming three homonuclear (As-As) bonds among tetra-arsenic triselenide As_4_Se_n_ entities (*n* = 3) with MCN = 2.57, these being *triangular-pyramidal* conformation I due to basal (As3) = (As-As-As) triangular neighboring with AsSe_3/2_ pyramid (see Figure 3e); open *chain*-like (As4) = (As-As-As-As) conformation II due to three homonuclear (As-As) bonds in *zig-zag* sequence (Figure 3f); and *star*-like (As3)As conformation III due to three homonuclear (As-As) bonds having a common origin on the fourth As atom (Figure 3g) [9,15]. In view of the calculated cluster-forming energies [9], the I-As_4_Se_3_ conformation, which is isostructural with the cage molecule in α-/β-modifications of dimorphite As_4_S_3_ [12,31,32,33], is the most favorable, approaching *E_f_* = 0.33 kcal/mol (see Table 1), which is less than in realgar-type x0-As_4_Se_4_ MFC, but more than in pararealgar-type x0-pr-As_4_Se_4_ MFC. The optimized configuration of this x0-I-As_4_Se_3_ MFC (see Figure 3e) [9] is composed of four small rings (3 pentagons, 1 triangle) by all seven atoms positioned at the surface of the same sphere in the under-constrained topology of the *C_3v_* symmetry (n_c_ = 2.71), resulting in a 0D structure with evident features of plastically crystalline phase (low calorimetric heat-transfer and strong thermal expansion responses) [27,34]. Other MFC iso-compositional to tetra-arsenic triselenide As_4_Se_3_ (such as x0-II-As_4_Se_3_ and x0-III-As_4_Se_3_) are unfavorable as compared with this x0-I-As_4_Se_3_ MFC (shown in Figure 3e), but they cannot be excluded from comprehensive consideration in view of their network derivatives, such as optimally constrained x1-1-II-As_4_Se_3_ NFC (n_c_ = 3.00), and over-constrained x3-II-As_4_Se_3_ and x3-III-As_4_Se_3_ NFC (both with n_c_ = 3.43, see Table 1).

The equilibrium between molecular and network-forming clustering is drastically disturbed in the transition to tetra-arsenic biselenide As_4_Se_n_ entities (n = 2) with MCN = 2.67, that is, at the border of the glass-forming region in the binary As-Se system [16,17]. Both parent MFC of this type (the x0-I-As_4_Se_2_ with n_c_ = 2.50 composed by (As-As) bond attached to As3 triangle, and x0-II-As_4_Se_2_ with n_c_ = 2.67 composed by four (As-As) bonds in *zig-zag* configuration) are unfavorable (see Table 1, and Figure 3h,i) [10]. The same is true of NFC derived from these MFC by breaking in one of two available Se atom positions. Conversely, the over-constrained x_2_-II-As_4_Se_2_ NFC derived by double x2-breaking (with *tetragon*-like As_4_ arrangement of four homonuclear As-As bonds) possesses *E_f_* = −0.72 kcal/mol. This NFC facilitates decomposition of the x0-I-As_4_Se_3_ MFC into realgar-type x0-As_4_Se_4_ MFC accompanied by the extraction of amorphous phase compositionally very close to As_4_Se_2_ [10].

In a group of tetra-arsenic monoselenide *thioarsenide*-type As_4_Se_n_ entities (n = 1) corresponding to MCN = 2.80, only two clusters are possible, with both x0-As_4_Se MFC (depicted in Figure 3j) and x1-As_4_Se NFC being under-constrained [10]. These clusters cannot be stabilized in realistic arsenoselenide conformations because of very unfavourable *E_f_* energies (see Table 1).

**Figure 5:**
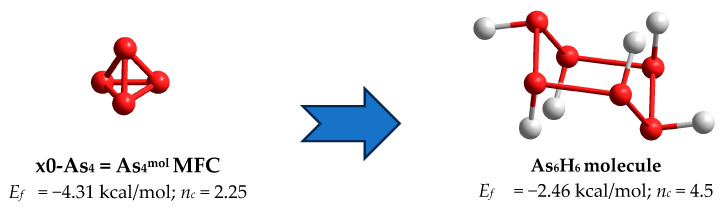
Ball-and-stick presentation of H-atom-saturated As_6_H_6_ molecular prototype of the As_6(2/3)_ = As_4_^net^ NFC in the form of a flattened pyramid-shaped unit (on the right) derived by distortion from parent x0-As_4_ = As_4_^mol^ tetrahedral-like MFC by breaking in one of three (As-As) bonds at each As atom. The terminated H atoms are grey-colored, As atoms are blue-colored, and chemical bonds between atoms are denoted by respectively colored sticks.

In a group of As_4_-type clusters restricting row of *thioarsenide*-type As_4_Se_n_ entities at n = 0 corresponding to *x* = 100 and MCN = 3.0 in the As_x_Se_100-x_ system, the molecular network balance is defined by two principal clusters characteristic of As polymorphs, these being the x0-As_4_ = As_4_^mol^ MFC in the form of a regular pyramid-shaped As_4_ tetrahedron (as shown in Figure 3k), and As_6(2/3)_ = As_4_^net^ NFC in the form of a flattened pyramid-shaped unit derived from As_4_^mol^ by breaking in one of three As-As bonds at each As atom within a two-dimensional double-layered network of chair-configurated 6-fold rings (see Figure 5) [10]. Within the arrangement of the three nearest neighbours, these clusters are differentiated by the calculated *E*_f_ forming energies, as shown in Table 1. The origin of network-type orthorhombic and rhombohedral As allotropes can be reasonably explained by distortion pathways beyond the three nearest neighbours [23,24,25,26], but this specificity cannot be accounted for in the current CINCA modelling. The under-constrained configuration of tetrahedral As_4_^mol^ MFC (having n_c_ = 2.25 in view of four small rings involved, 4 triangles) appears to be very unfavourable, resulting in the *E_f_* forming energy approaching only −4.31 kcal/mol, thus confirming the γ-As phase stabilization exceptionally in a gaseous state [25,26]. In contrast, the over-constrained configuration of As_4_^net^ NFC (having n_c_ = 4.5 in view of only one small ring involved, 1 hexagon) composing a double-layer honeycomb structure of chair-configurated As_6(2/3)_ rings typical for rhombohedral (grey or metallic α-As) and orthorhombic (black or semiconducting β-As) allotropes [23,24,25,26] are more promising in crystallization processes, resulting in *E_f_*~ −2.46 kcal/mol (see Table 1). This explains the appearance of rhombohedral α-As phase as the most stable allotropic modification under nanostructurization-driven transformations in melt-quenched As-Se alloys [10].

Within the compositional map of molecular network clustering in the As_x_Se_100-x_ alloys covering a full row of *thioarsenide*-type As_4_Se_n_ entities (0 ≤ n ≤ 6) reproduced in Figure 1, we can distinguish between a molecular-forming counter line connecting the settle-points of the most favorable MFC (shown as dotted red-colored line) and a network-forming counter line connecting the settle-points of the most favorable NFC (depicted as dotted blue-colored line in Figure 1). By comparing these compositional counter lines, it can be seen that the network-crystalline conformations prevail at the boundary of the As_4_Se_n_ row for n = 6 (As_4_Se_6_) and n = 0 (As_4_), and exceptionally for n = 2 (As_4_Se_2_), while *thioarsenides* inside this row for n = 5 (As_4_Se_5_), n = 4 (As_4_Se_4_), and n = 3 (As_4_Se_3_) are dominated by a molecular-forming trend. It is worth noting that both MFC and NFC with n = 1 (As_4_Se) are very unfavorable with respect to other *thioarsenides*.

In full harmony with these findings, the boundary *thioarsenides* labeled in Figure 1 by open red circles possess the network-crystalline conformations at n = 6 (due to x4-As_4_Se_6_ = As_2_Se_3_^net^ NFC, the basic structural motives of monoclinic As_2_Se_3_ [21,22]) and n = 0 (due to x0-As_6(2/3)_ = As_4_^net^ NFC, the basic structural motives of network As polymorphs, the rhombohedral α-As or orthorhombic β-As [23,24,25,26]), and molecular-crystalline conformations at n = 4 (due to x0-As_4_Se_4_ MFC, the basic motives of monoclinic As_4_Se_4_ [18,19,20]) and n = 3 (due to x0-I-As_4_Se_3_ MFC, the basic structural motives of As_4_Se_3_ allotropes [27]). Thus, the boundary crystalline entities terminating the *thioarsenides* As_4_Se_n_ row at n = 6 and n = 0 possess the better network-forming ability, while a molecular-crystalline tendency prevails inside this *thioarsenides* row at n = 4 and n = 3.

### 2.2. Inter-Phase Equilibria in Over-Stoichiometric Arsenoselenides As_x_Se_100−x_ (40 ≤ x ≤ 100) Governed by Molecular Network Clustering

The coexistence of several stable crystalline phases in the As_x_Se_100-x_ alloys (40 ≤ x ≤ 100), covering a full row of As_4_Se_n_ *thioarsenides* (0 ≤ n ≤ 6)—two of which are of network-type, terminating this row by boundary entities with n = 6 (As_2_Se_3_^net^ equivalent to As_4_Se_6_) and n = 0 (As_4_^net^); and two others of which are molecular-type, forming an intrinsic part of this row at n = 4 (As_4_Se_4_^mol^) and n = 3 (As_4_Se_3_^mol^)—is defined by primary full equilibrium line 1 connecting the respective settle-points corresponding to the forming energies (*E*_f_) of these most favorable (regular) crystalline entities [As_2_Se_3_^net^–As_4_Se_4_^mol^–As_4_Se_3_^mol^–As_4_^net^]. In reality, equilibrium line 1 is composed of three cut-sections, these being (i) left-terminated network–molecular [As_2_Se_3_^net^–As_4_Se_4_^mol^], (ii) intrinsic molecular–molecular (As_4_Se_4_^mol^–As_4_Se_3_^mol^), and (iii) right-terminated molecular–network [As_4_Se_3_^mol^–As_4_^net^].

Because of the sharp drop in the *E*_f_ energy for As_4_^net^ NFC (see Table 1), a few cut-section lines corresponding to partial inter-crystalline equilibria can be derived from this primary equilibrium line 1, such as primary partial equilibrium line 3 for regular boundary–boundary *thioarsenide* entities [As_2_Se_3_^net^–As_4_^net^], and lines 3.1 and 3.2 for regular intrinsic-boundary entities [As_4_Se_4_^mol^–As_4_^net^] and [As_4_Se_3_^mol^–As_4_^net^], respectively. The cross-point of any of these primary (full or partial) equilibrium lines with respective *thioarsenide* compositional line (defined by *n* parameter or MCN value) corresponds to a simple mixture of these boundary regular phases, the amounts of which are inversely proportional to the distances to the boundary points.

Thus, the *thioarsenide*-type entities (NFC and MFC) positioned at the molecular network clustering map below the primary full equilibrium line 1 [As_2_Se_3_^net^−As_4_Se_4_^mol^−As_4_Se_3_^mol^−As_4_^net^] represent amorphous substances that can be stabilized by decomposition on the regular phases positioned most closely along this line. Respectively, the primary partial equilibrium lines 3, 3.1, and 3.2 define amorphous substances, which can be stabilized by decomposition on a mixture of respective boundary *thioarsenide*-type phases.

In a similar manner, a set of secondary equilibrium lines can be introduced for *thioarsenide*-type entities that are competitive to most favorable (regular) ones. The first of such irregular entities corresponds to the As_4_^mol^ MFC, which possesses a smaller *E*_f_ energy than iso-compositional As_4_^net^ NFC (see Table 1). Therefore, the secondary equilibrium line 4 [As_2_Se_3_^net^−As_4_^mol^] connecting the settle-points of regular network-type As_2_Se_3_^net^ and irregular molecular-type As_4_^mol^ entities can be introduced to define a mixture of both components. By analogy, the secondary partial equilibrium line 4.1 for a mixture of regular As_4_Se_4_^mol^ and irregular As_4_^mol^ entities and secondary partial equilibrium line 4.2 for a mixture of regular As_4_Se_3_^mol^ and irregular As_4_^mol^ entities can be reconstructed on the clustering map (Figure 1). The cross-point of any of these secondary equilibrium lines with the respective *thioarsenide* composition (defined by the *n* parameter) corresponds to a mixture of both boundary phases.

The absence of regular crystalline structures corresponding to the x0-As_4_Se_6_ MFC (labeled as As_4_Se_6_^mol^ in Figure 1) does not mean an absence of these *thioarsenide*-type structural entities in amorphous arsenoselenides derived in more non-equilibrium technological conditions or under nanostructurization influence [11]. That is why the secondary equilibrium line 2 [As_4_Se_6_^mol^−As_4_^mol^] connecting the settle-points of both irregular molecular boundary entities terminating the *thioarsenides* As_4_Se_n_ row at *n* = 6 and 0 can be introduced. This equilibrium line 2 (As_4_Se_6_^mol^−As_4_^mol^) defines a mixture of both irregular molecular *thioarsenide*-type phases, which can be extracted in arsenoselenide compounds in amounts inversely proportional to the distances to the respective boundary points.

### 2.3. Disproportionality Analysis of Native and Mechanoactivated Molecular Network Conformations in Over-Stoichiometric As_x_Se_100−x_ Arsenoselenides (40 ≤ x ≤ 100)

Crystallization processes in amorphous alloys can be essentially complicated by the accompanying decomposition reactions [35,36,37,38]. Thus, crystallization without compositional changes (also known as polymorphous crystallization [36]) occurs if the free energy of supersaturated crystalline alloy is lower than that of amorphous alloy [36]. The primary crystallization in one of the stable boundary phases occurs if the concentration of amorphous substance shifts until the stable phase stops crystallizing by reaching the metastable equilibrium. At last, if simultaneous decomposition of amorphous alloy into two stable phases occurs with the greatest driving force, then eutectoid crystallization can be realized by discontinuous reaction in a whole range of compositions.

Similar processes are expected in amorphous chalcogenide alloys prepared by conventional melt quenching [5], in part, the *thioarsenide*-type As_4_Se_n_ alloys. Thus, more than four decades ago, Blachnik and Wickel [27] recognized the thermal behaviour of A_4_B_3_ cage-like molecules (A = P, As; B = S, Se) in plastically and normally crystalline modifications of these A_4_B_3_ polymorphs. In their suggestion [27], these chalcogenide alloys decompose peritectoidally into A_4_B_4_ molecules and unidentified amorphous substance.

In molecular network alloys like binary arsenoselenides, inter-phase equilibria lead towards *polymorphic* and *polyamorphic* transitions in the vicinity of some *thioarsenide*-type As_4_Se_n_ compositions, especially under mechanoactivated nanostructurization [8,9,10,11].

Disproportionality analysis employing the map of molecular network atomic clustering in the As-Se system (Figure 1) serves as a key to understanding the essence of such processes. Within this map, each NFC contributing to amorphization can be presented by the settle-point below the network-forming counter line (bold blue dotted line in Figure 1). The polymorphous crystallization with negative energetic barrier can be realized in this alloy as a spontaneous transition into a crystalline state along a vertical compositional line. In boundary arsenoselenides terminating the row of *thioarsenides* As_4_Se_n_ (for *n* = 6 and *n* = 0), crystallization prevails by transition into the most favorable state of x4-As_4_Se_6_ = As_2_Se_3_^net^ and As_4_^net^ NFC, having the best cluster-forming energies *E_f_* as compared with iso-compositional x0-As_4_Se_6_ = As_4_Se_6_^mol^ and x0-As_4_ = As_4_^mol^ MFC. In arsenoselenides inside the As_4_Se_n_ row (for *n* = 4 and *n* = 3), polymorphous crystallization prevails by transition into most the favorable state of x0-As_4_Se_4_ = As_4_Se_4_^mol^ and x0-As_4_Se_3_ = As_4_Se_4_^mol^ MFC, having the best *E_f_* energy as compared with iso-compositional x1-As_4_Se_4_ and x1-1-II-As_4_Se_3_ NFC.

It is worth noting that if the NFC settle-point is located below one of the equilibrium lines defined by some boundary arsenoselenide compounds, these NFC are metastable undergoing spontaneous decomposition into a mixture of these boundary compounds. Decomposition in As_6(2/3)_ = As_4_^net^ NFC contributes to primary crystallization of regular As phase (the rhombohedral α-As [23,24]), while x0-As_4_ = As_4_^mol^ MFC contribute to the irregular amorphous phase of this alloy. Thus, with respect to the clustering map (see Figure 1), the As_4_Se_n_ conformations based on NFC with *E*_f_ energies above the secondary equilibrium line 4 (As_2_Se_3_^net^−As_4_^mol^) connecting the settle-points corresponding to the most favorable x4-As_4_Se_6_ = As_2_Se_3_^net^ NFC and competitive x0-As_4_Se_4_ = As_4_Se_4_^mol^ MFC serve as principal building blocks for amorphous arsenoselenides, which can be fabricated by conventional melt quenching.

Let us analyze the expected *amorphization/reamorphization* scenarios in these alloys, covering a full row of *thioarsenide*-type As_4_Se_n_ entities (0 ≤ n ≤ 6).

In the point of stoichiometry (MCN = 2.40), the most favorable x4-As_4_Se_6_ = As_2_Se_3_^net^ NFC composed of two corner-sharing AsSe_3/2_ pyramids in optimally constrained geometry (*n*_c_ = 3.00) are preferred over parent x0-As_4_Se_6_ = As_4_Se_6_^mol^ MFC (see Table 1), determining the network nature of both crystalline and amorphous *thioarsenide*-type polymorphs [6,7,11].

With the transition to As_4_Se_5_ *thioarsenides*, polymorphous crystallization into one stable phase is suppressed by competitive amorphization due to the x4-As_4_Se_5_ NFC with *E*_f_ energy above the secondary equilibrium line 4 [As_2_Se_3_^net^−As_4_^mol^] (see Figure 1). At this point (MCN = 2.44), decomposition into the nearest regular phases (based on As_2_Se_3_^net^ NFC and As_4_Se_4_^mol^ MFC) results in their mixture with *E*_f_ = 0.346 kcal/mol, which is 0.026 kcal/mol better than in the x0-As_4_Se_5_ MFC [11]. This means that just-formed x0-As_4_Se_5_ MFC will be spontaneously decomposed on these boundary entities without stabilization of the crystalline phase.

At the point of the As_4_Se_4_-type *thioarsenides* (MCN = 2.50), the known molecular-crystalline counterpart (which is monoclinic As_4_Se_4_ [18,19,20]) is preferred due to the x0-As_4_Se_4_ = As_4_Se_4_^mol^ MFC of the best cluster-forming energy among all *thioarsenide*-type entities (*E_f_* = 0.40 kcal/mol, see Table 1) [8]. The competitive NFC of this type (x1-As_4_Se_4_, x4-As_4_Se_4_, x3-As_4_Se_4_, x4-pr-As_4_Se_4_, x1-1-pr-As_4_Se_4_, x2-1-As_4_Se_4_, x3-3-pr-As_4_Se_4_) are tightly and uniformly grouped on the clustering map above the secondary equilibrium line 4 [As_2_Se_3_^net^−As_4_^mol^], approaching the settle-point of As_4_Se_4_^mol^ MFC but not overcoming it (Figure 1). This speaks in a favor of high crystallization ability of this arsenoselenide alloy. Moreover, because of the NFC positioned on the clustering map above the secondary equilibrium line 4 [As_2_Se_3_^net^−As_4_^mol^] and primary partial equilibrium line 3 [As_2_Se_3_^net^−As_4_^net^], nanomilling-driven amorphization and re-amorphization processes in this alloy can also be facilitated, resulting in stabilization of both molecular- and network-type As polymorphs based on As_4_^mol^ MFC and As_4_^net^ NFC.

In As_4_Se_3_-type *thioarsenides* (MCN = 2.57), molecular network disproportionality is highly disturbed. Polymorphous crystallization of this alloy into orthorhombic α’-As_4_Se_3_ phase dominates because of the preference of dimorphite-type x0-I-As_4_Se_3_-As_4_Se_3_^mol^ MFC, possessing very promising *E_f_* energy, approaching 0.33 kcal/mol (see Table 1) [9]. The NFC contributing to the amorphization of this alloy are located in the narrow interval above the secondary equilibrium line 4 [As_2_Se_3_^net^−As_4_^mol^] and below the primary partial equilibrium line 3 [As_2_Se_3_^net^−As_4_^net^] (see Figure 1). There are no As_4_Se_3_-type NFC closely approaching the x0-I-As_4_Se_3_-As_4_Se_3_^mol^ MFC. This means that direct crystallization of this alloy from the NFC state is rather impossible, in view of the competitive decomposition of these entities within the nearest equilibrium lines. Spontaneous decomposition of these NFC prevails along the primary partial equilibrium line 3 [As_2_Se_3_^net^−As_4_^net^] and secondary partial equilibrium line 4.1 [As_4_Se_4_^mol^−As_4_^mol^], resulting in both As polymorphs based on As_4_^mol^ MFC and As_4_^net^ NFC, whereas induced decomposition along secondary equilibrium line 4 [As_2_Se_3_^net^−As_4_^mol^] results in amorphous As based on As_4_^mol^ MFC.

As follows from the clustering map (Figure 1), the x2-II-As_4_Se_2_ NFC (which can be derived from the As_4_Se_2_-II molecule by double breaking in all available Se atom positions) is the last suitable candidate introducing network *polyamorphism* in this *thioarsenide* alloy [10]. Both parent MFC related to As_4_Se_2_ stoichiometry (x0-I-As_4_Se_2_ and x0-II-As_4_Se_2_) are very unfavorable, but the x2-II-As_4_Se_2_ NFC is prone to spontaneous decomposition along the secondary partial equilibrium line 4.2, and primary partial equilibrium line 3.1 and 3.2, resulting in regular *thioarsenide*-type phases (based on the most favorable As_4_Se_4_^mol^ and As_4_Se_3_^mol^ MFC and As_4_^net^ NFC). However, complete eutectoid crystallization of this alloy is rather prohibited by the positive barrier of competitive induced decomposition along the primary partial equilibrium line 3 [As_2_Se_3_^net^−As_4_^net^]. The former spontaneous decomposition processes explain the location of the glass-forming border in the As-Se system near the As_4_Se_2_ composition, while the latter over-barrier decomposition processes are responsible for the nanostructurization-driven *re-amorphization* in this alloy [10].

With further increase in As content towards tetra-arsenic monoselenide As_4_Se stoichiometry (MCN = 2.80), a complete breakdown in forming ability occurs for iso-compositional MFC and NFC, presumably because of unrealistic steric constraints limiting the stabilization of As_4_Se entities [10]. Only products of eutectoid decomposition on the most favorable *thioarsenide*-type entities (mainly As_4_Se_3_^mol^ and As_4_^net^) can be stabilized in this alloy.

Disproportionality analysis of *thioarsenide*-type As_4_Se_n_ conformations is reasonably completed in the As polymorphs [23,24,25,26] by preference of the As_6(2/3)_ = As_4_^net^ NFC in a form of chair-configurated 6-fold rings over the x0-As_4_ = As_4_^mol^ MFC in a form of pyramid-shaped As_4_ tetrahedra. From most principal viewpoints, the diversity of decomposition reactions in over-stoichiometric arsenoselenides covering a full row of As_4_Se_n_ entities (0 ≤ n ≤ 6) is governed by this anomaly in their boundary conformations.

## 3. Methods

### 3.1. Cluster Modeling of Molecular Conformations in Covalent-Bonded Substances

The optimized conformations of *thioarsenide*-type As_4_Se_n_ molecular entities (MFC and NFC) were reconstructed employing ab initio quantum-chemical modeling with cluster-simulation code CINCA (cation-interlinked network cluster approach) [28,29]. The HyperChem Release 7.5 program package based on the restricted Hartree–Fock self-consistent field method with split-valence double-zeta basis set and single polarization function 6-311G* [39,40,41] was used. The geometrical optimization and single-point energy calculations were performed by the Fletcher–Reeves conjugate gradient method until a root-mean-square gradient of 0.1 kcal/(Å·mol) was reached. For comparison within the As-Se family, the final average cluster-forming energies were averaged for all constituent atoms in the molecule *E_f_* = (*E*_f_^Σ^)^av^ and recalculated with respect to the forming energy of a single trigonal AsSe_3/2_ pyramid (*E_f_* = −72.309 kcal/mol [29]).

The CINCA modeling [28,29] allows simulation of molecular-type conformations in solid systems possessing full saturation of covalent bonding like the As_x_Se_100-x_ alloys characterized by different numbers of covalent chemical bonds per atom (MCN). To compare the clusters, accounting for small rings characteristic of molecular *thioarsenide*-type structures, the average number of the Lagrangian constraints (*n_c_*) associated with stretching and bending forces ascribed to intra-molecular bonds within the cluster was recalculated using the Phillips–Thorpe constraint-counting algorithm [42,43,44].

### 3.2. Disproportionality Analysis of Molecular Network Conformations in Chalcogenide Alloys

Disproportionality analysis of molecular network conformations foresees energetic differentiation between all possible *thioarsenide*-type As_4_Se_n_ molecules (nominated as parent MFC) and molecular prototypes of iso-compositional NFC derived from these MFC by all possible breaks on separate homonuclear (Se_1/2_…Se_1/2_) fragments in available Se atom positions [28,29]. The self-consistent molecular configurations of the NFC were reconstructed by saturating the Se dangling bonds with the hydrogen (H) atoms, with low bonding energy in the covalent structures (∼3 kcal/mol [41,45,46]). This termination procedure is also quite reasonable since the electronegativity of terminated H atoms (2.20) is close to those of other atoms in As-Se conformations, being intermediate between the electronegativities of the As and Se atoms (respectively approaching 2.18 and 2.55 [47]). Therefore, there are no strong disturbances in electron density distribution within the NFC in chalcogenide compounds due to terminated H atoms, transforming these network entities (NFC) into self-consistent molecular prototypes.

## 4. Conclusions

Molecular network conformations in over-stoichiometric As-bearing arsenoselenides As_x_Se_100−x_ (40 ≤ x ≤ 100) covering a full row of *thioarsenide*-type As_4_Se_n_ entities (0 ≤ n ≤ 6) are comprehensively and systematically analyzed by ab initio quantum-chemical modeling employing the cluster-simulation code CINCA (cation-interlinked network cluster approach). Native (melt-quenching-derived) and nanostructurization-driven (activated by high-energy milling) *polymorphic* and *polyamorphic* transformations initiated by the decomposition of *thioarsenide*-type As_4_Se_n_ cage-like molecules and incorporation of their remnants into a newly polymerized arsenoselenide network are identified on the map of molecular network clustering in an As-Se sysyem. Within this map reconstructed for over-stoichiometric arsenoselenides within a full row of thioarsenide-type As_4_Se_n_ entities (0 ≤ n ≤ 6), the compositional counter lines corresponding to preferential molecular- or network-forming tendencies in a system are determined, showing that network-crystalline conformations prevail in the boundary As_4_Se_n_ compositions (n = 6 and 0), while molecular-crystalline conformations dominate inside the As_4_Se_n_ row (n = 4 and 3). A set of primary and secondary equilibrium lines is introduced in the clustering map to account for inter-phase equilibria between the most favorable (regular) and competitive (irregular) *thioarsenide* phases. Straightforward interpretation of decomposition reactions accompanying induced crystallization and amorphization (reamorphization) processes in the arsenoselenides is achieved employing disproportionality analysis of *thioarsenide*-type molecular network conformations within the developed clustering map. Bifurcation of molecular network clustering at the boundaries of the *thioarsenides* As_4_Se_n_ row is shown to disturb the inter-phase equilibria inside this row, leading to many unexpected consequences, such as the absence of stable tetra-arsenic pentaselenide (As_4_Se_5_) molecular-crystalline species; polyamorphism in mechanoactivated *thioarsenide*-type As_4_Se_n_ alloys; breakdown in the glass-forming ability of melt-quenching-derived arsenoselenides in the vicinity of tetra-arsenic biselenide (As_4_Se_2_) composition; plastically and normally crystalline polymorphism in tetra-arsenic triselenide (As_4_Se_3_) thioarsenides, and so on.

## Figures and Tables

**Table 1:** Parameterization of *thioarsenide*-type As_4_Se_n_ MFC and their network derivatives (NFC) in over-stoichiometric arsenoselenides As_x_Se_100−x_ (the cluster-forming energies (E_f_^Σ^)^av^ are defined with respect to the energy of a single AsSe_3/2_ pyramid, E_f_(AsSe_3/2_) = −72.309 kcal/mol [29]).

MCN; n	*Thioarsenide*-Type As_4_Se_n_ MFC		*Thioarsenide*-Type As_4_Se_n_ NFC	
	MFC Nomenclature; n_c_; Small Rings	*E*_f_, kcal·mol^−1^	NFC Nomenclature; n_c_; Separate Units, and/or Small Rings	*E*_f_, kcal·mol^−1^
2.40; n = 6	x0-As_4_Se_6_ = As_4_Se_6_^mol^; n_c_ = 3.00; 4 hexagons	−0.67	x4-As_4_Se_6_ = As_2_Se_3_^net^; n_c_ = 3.00; 2 separate units, no small rings	0.31
2.44; n = 5	x0-As_4_Se_5_; n_c_ = 2.89; 2 hexagons, 2 pentagons	0.32	x1-1-As_4_Se_5_; n_c_ = 2.89; 2 pentagons	−1.01
			x1-2-As_4_Se_5_; n_c_ = 3.00; 1 pentagon, 1 hexagon	−0.37
			x2-1-As_4_Se_5_; n_c_ = 3.00; 1 pentagon	−6.07
			x2-2-As_4_Se_5_; n_c_ = 3.11; no small rings	−8.02
			x2-3-As_4_Se_5_; n_c_ = 3.11; 1 hexagon	−0.60
			x3-1-As_4_Se_5_; n_c_ = 3.11; no small rings	0.16
			x3-2-As_4_Se_5_; n_c_ = 3.00; 2 separate units, 1 pentagon	0.05
			x4-As_4_Se_5_; n_c_ = 3.11; 2 separate units, no small rings	0.22
			x5-As_4_Se_5_; n_c_ = 3.11; 3 separate units, no small rings	0.05
2.50; n = 4	x0-As_4_Se_4_; n_c_ = 2.875; 4 pentagons, 4 hexagons	0.40	x1-As_4_Se_4_; n_c_ = 3.00; 2 pentagons, 1 hexagon	0.25
			x2-1-As_4_Se_4_; n_c_ = 3.125; 1 pentagon	−0.42
			x2-2-As_4_Se_4_; n_c_ = 3.25; 1 hexagon	−9.64
			x3-As_4_Se_4_; n_c_ = 3.25; no small rings	0.05
			x4-As_4_Se_4_; n_c_ = 3.25; 2 separate units, no rings	0.11
	x0-pr-As_4_Se_4_; n_c_ = 2.75; 1 hexagon, 2 pentagons, 1 tetragon	0.30	x1-1-pr-As_4_Se_4_; n_c_ = 3.00; 2 pentagons	−0.25
			x1-2-pr-As_4_Se_4_; n_c_ = 2.875; 1 pentagon, 1 tetragon	−1.39
			x1-3-pr-As_4_Se_4_; n_c_ = 3.00; 1 hexagon, 1 tetragon	−1.55
			x2-1-pr-As_4_Se_4_; n_c_ = 3.00; 1 tetragon	−4.93
			x2-2-pr-As_4_Se_4_; n_c_ = 3.125; 1 pentagon	−7.36
			x2-3-pr-As_4_Se_4_; n_c_ = 3.00; 1 tetragon	−9.78
			x2-4-pr-As_4_Se_4_; n_c_ = 3.25; 1 hexagon	−5.66
			x3-1-pr-As_4_Se_4_; n_c_ = 3.00; 2 separate units, 1 tetragon	−0.85
			x3-2-pr-As_4_Se_4_; n_c_ = 3.25; no small rings	−2.81
			x3-3-pr-As_4_Se_4_; n_c_ = 3.25; no small rings	−0.47
			x4-pr-As_4_Se_4_; n_c_ = 3.25; 2 separate units, no rings	−0.03
2.57; n = 3	x0-I-As_4_Se_3_; n_c_ = 2.71; 3 pentagons, 1 triangle	0.33	x1-I-As_4_Se_3_; n_c_ = 2.86; 1 pentagon + 1 triangle	−1.90
			x2-I-As_4_Se_3_; n_c_ = 3.00; 1 triangle	−9.13
			x3-I-As_4_Se_3_; n_c_ = 3.00; 2 separate units, 1 triangle	−1.47
	x0-II-As_4_Se_3_; n_c_ = 2.71; 2 pentagons, 2 tetragons	−0.94	x1-1-II-As_4_Se_3_; n_c_ = 3.00; 1 tetragon, 1 pentagon	−0.60
			x1-2-II-As_4_Se_3_; n_c_ = 2.86; 2 tetragons	−2.36
			x2-1-II-As_4_Se_3_; n_c_ = 3.29; 1 pentagon	−8.76
			x2-2-II-As_4_Se_3_; n_c_ = 3.14; 1 tetragon	−5.80
			x3-II-As_4_Se_3_; n_c_ = 3.43; no small rings	−0.60
	x0-III-As_4_Se_3_; n_c_ = 2.57; 1 hexagon, 3 tetragons	−2.44	x1-III-As_4_Se_3_; n_c_ = 2.86; 2 tetragons	−3.22
			x2-III-As_4_Se_3_; n_c_ = 3.14; 1 tetragon	−1.29
			x3-III-As_4_Se_3_; n_c_ = 3.43; no small rings	−0.88
2.67; n = 2	x0-I-As_4_Se_2_; n_c_ = 2.50; 1 pentagon, 2 tetragons, 1 triangle	−3.59	x1-I-As_4_Se_2_; n_c_ = 2.83; 1 tetragon, 1 triangle	−3.27
			x2-I-As_4_Se_2_; n_c_ = 3.17; 1 triangle	−4.42
	x0-II-As_4_Se_2_; n_c_ = 2.67; 5 tetragons	−4.42	x1-II-As_4_Se_2_; n_c_ = 3.00; 3 tetragons	−3.46
			x2-II-As_4_Se_2_; n_c_ = 3.33; 1 tetragon	−0.72
2.80; n = 1	x0-As_4_Se; n_c_ = 2.40; 2 tetragons, 2 triangles	−5.20	x1-As_4_Se; n_c_ = 2.80; 2 triangles, 1 tetragon	−8.30
3.00; n = 0	x0-As_4_ = As_4_^mol^; n_c_ = 2.25; 4 triangels	−4.31	As_4_^net^; n_c_ = 4.50; 1 hexagon	−2.46

## Data Availability

The original contributions presented in this study are included in the article. Further inquiries can be directed to the corresponding author.
